# Effects of Resistance Exercise Applied Early After Coronary Artery Bypass Grafting: a Randomized Controlled Trial

**DOI:** 10.5935/1678-9741.20150077

**Published:** 2015

**Authors:** Nayana Nazaré Pessoa Sousa Ximenes, Daniel Lago Borges, Reijane Oliveira Lima, Mayara Gabrielle Barbosa e Silva, Luan Nascimento da Silva, Marina de Albuquerque Gonçalves Costa, Thiago Eduardo Pereira Baldez, Vinícius José da Silva Nina

**Affiliations:** 1 Hospital Universitário da Universidade Federal do Maranhão (HUUFMA), São Luís, MA, Brazil.; 2 Programa de Pós-Graduação em Saúde do Adulto e da Criança, UFMA, São Luis, MA, Brazil.

**Keywords:** Exercise, Rehabilitation, Myocardial Revascularization

## Abstract

**OBJECTIVE:**

To evaluate the effects of resistance exercise applied early after coronary artery bypass grafting.

**METHODS:**

It is a randomized controlled trial with 34 patients undergoing coronary artery bypass grafting between August 2013 and May 2014. Patients were randomized into two groups by simple draw: a control group (n=17), who received conventional physical therapy and an intervention group (n=17), who received, additionally, resistance exercise. Pulmonary function and functional capacity were evaluated in preoperative period and hospital discharge by spirometry and the six-minute walk test. For statistical analysis, we used the following tests: Shapiro-Wilk, Mann-Whitney, Student's *t* and Fisher's exact. Variables with *P*<0.05 were considered significant.

**RESULTS:**

Groups were homogeneous in terms of demographic, clinical and surgical variables. Resistance exercise exerted no effect on pulmonary function of intervention group compared to control group. However, intervention group maintained functional capacity at hospital discharge measured by percentage of predict distance in 6MWT (54.122.7% *vs*. 52.515.5%, *P*=0.42), while control group had a significant decrease (59.211.1% *vs.* 50.69.9%, *P*<0.016).

**CONCLUSION:**

Our results indicate that resistance exercise, applied early, may promote maintenance of functional capacity on coronary artery bypass grafting patients, having no impact on pulmonary function when compared to conventional physical therapy.

**Table:** 

**Abbreviations, acronyms & symbols**	
6MWT	= Six-minute walk test
ACSM	= American College of Sports Medicine
AHA	= American Heart Association
BMI	= Body mass index
CABG	= Coronary artery bypass grafting
FEV1	= Forced expiratory volume in the first second
FEV1/FVC	= Forced expiratory coefficient in the first second
FVC	= Forced vital capacity
ICU	= Intensive care unit
LL	= Lower limbs
PEF	= Peak expiratory flow
UL	= Upper limbs

## INTRODUCTION

Coronary artery bypass grafting (CABG) is the main treatment for coronary artery disease when medicines or percutaneous procedures are not enough to revert symptoms^[1,2]^. Over the past 10 years, the use of more durables grafts, aiming to minimize injuries to the patients, brought a progressive improvement to this surgical procedure^[3]^.

This progress has also encouraged advancements in the area of cardiac rehabilitation, which uses supervised exercise as therapeutic basis^[4,5]^. It is known that the practice of regular physical activity is beneficial and recommended as one of the best treatments for patients undergoing cardiac surgery, intending to reduce the risk of future cardiac events^[6]^.

Therefore, cardiac rehabilitation protocols have been developed to restore patients' daily activities, emphasizing physical and educational activities aiming lifestyle changes. Recent therapeutic techniques allow early hospital discharge with minimal reduction of functional capacity^[5,7]^.

In this respect, these patients have performed not only aerobic exercises but also resistance exercises and this practice is recommended for increase in functional capacity. The benefits of resistance exercise associated to aerobic exercise include an overall decrease of recurrent cardiac events, increased survival, physical and psychosocial independence, and improved quality of life^[8]^.

Since the first guidelines about resistance training established in 2000 by American Heart Association (AHA) and American College of Sports Medicine (ACSM), this exercise modality has become more accepted and included in exercise programs for subjects with and without cardiovascular disease. Brazilian Guidelines for Cardiac Rehabilitation and the ACSM report that muscle strength is fundamental to health, to the maintenance of functional capacity, and to achievement of satisfactory quality of life^[5,9]^.

Considering the above, our aim consists in evaluate the effects of resistance exercise applied early after coronary artery bypass grafting on functional capacity and pulmonary function.

## METHODS

### Type and location of the study

This randomized clinical trial was conducted in a Brazilian university hospital.

### Study sample

A non-probabilistic sample of adult patients undergoing CABG between August 2013 and May 2014 were included in our study. Patients not included had chronic obstructive pulmonary disease, neurological sequelae, neuromuscular disease or submitted to associated, emergency or off-pump surgery. Perioperative deaths, surgical re-intervention and patients who remained on mechanical ventilation longer than 24 hours or noninvasive ventilation longer than four hours were excluded.

**Fig. 1:**
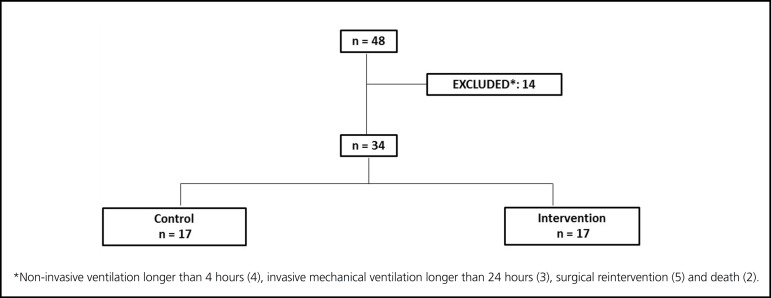
Flow chart

Total sample consisted of 34 patients randomized by simple draw into two groups: a control group with 17 patients undergoing conventional physical therapy and an intervention group with 17 patients undergoing resistance exercise (Figure 1).

### Patient evaluation

In preoperative period, patients were informed and guided about the study and were subsequently evaluated. Data were collected from medical records and physical therapy evaluation form using a specific instrument developed for the study divided into three parts, containing data about pre-, intra-, and postoperative periods. Then, to assess pulmonary function, we performed spirometry, which involved the measurement forced vital capacity (FVC), forced expiratory volume in the first second (FEV1), forced expiratory coefficient in the first second (FEV1/ FVC [%]), and peak expiratory flow (PEF), following criteria established by the guidelines for pulmonary function tests^[10]^. For mortality risk assessment we used EuroScore II^[11]^.

The six-minute walk test (6MWT) was performed to evaluate functional capacity according to guidelines of American Thoracic Association^[12]^. The equation proposed by Iwama et al.^[13]^ was applied to estimate the predicted distance walked in the 6MWT. We performed spirometry and 6MWT preoperatively and at hospital discharge.

### Proposed treatments

Patients included in control group received conventional physical therapy consisting of diaphragmatic breathing exercises and progressive ambulation, varying according to postoperative day and subjective tolerance to exercise assessed by Borg scale^[14]^. Additionally, were performed assisted and active exercises for upper (UL) and lower limbs (LL).

Intervention group received diaphragmatic breathing exercises, progressive ambulation and resistance exercises for UL and LL. Initial load was established using a stress test, which consisted of a set of 10 repetitions using 0.5 kg dumbbells for elbow flexion and 1.0 kg shin pads for knee extension. The load was defined by subjective perceived effort using Borg scale^[6,14]^.

Resistance exercises consisted of muscle training with dumbbells for UL (biceps and triceps) and shin pads for LL (quadriceps) with the patient in Fowler 45, during immediate postoperative period, and subsequently sitting in bed with free LL allowing alternated and unilateral knee extension. Training of hip adductors and abductors and triceps surae occurred at ward, where patient was free to ambulate, and consisted of three sets of 10 repetitions for each muscle group. Sessions were performed right after extubation in both the groups, for 30 minutes, twice a day in the intensive care unit (ICU) and once a day at ward until hospital discharge.

During exercises in the ICU, blood pressure, heart and respiratory rates, and oxygen saturation were monitored using a multiparameter monitor model Infinity Delta XL (Dräger Medical, Lübeck, Germany). At ward, before and after each session, blood pressure was measured using a digital sphygmomanometer (Panasonic model EW-BW30S, São Paulo, Brazil). Heart rate and oxygen saturation were monitored with a digital oximeter (Acc U Rate, United States).

### Statistical analysis

Data collected were statistically analyzed using software Stata/SE 12.1 (Stata Corp, College Station, Texas, USA). To identify normality Shapiro-Wilk test was used. Quantitative variables were expressed as mean and standard deviation and their differences assessed using Mann-Whitney test for non-normal variables and Student's t test for normal variables (independent and paired). For categorical variables, Fisher's exact test was used. The results were considered statistically significant when *P*<0.05.

### Ethical aspects

In compliance with Resolution 466/12 of the National Health Council, the present study was approved by the Institutional Research Ethics Committee (No. 337.227).

## RESULTS

In our study were included 48 patients. Fourteen were excluded due non-invasive ventilation longer than four hours (n=4), mechanical ventilation longer than 24 hours (n=3), surgical reintervention (n=5) and death (n=2) (Figure 1). Mortality rate in this population was 4.2%.

Therefore, the final sample consisted of 34 patients, predominantly men (76.5%), mean age of 60.96.8 years, body mass index (BMI) of 25.73.3 kg/m^2^, and EuroScore II of 0.760.14%. Groups were homogeneous in terms of demographic, clinical, and surgical characteristics (Table 1).

**Table 1:** Demographic, clinical, surgical and postoperative data, per group, of patients undergoing CABG

**Variables**	**Intervention (n = 17)**	**Control (n = 17)**	**Total (%)**	***P***
**Gender**				0.69^a^
Male	14	12	26 (76.5)	
Female	3	5	8 (23.5)	
**Age (years)**	59.9±7	61.8±6.7	60.9±6.8	0.43^b^
**BMI (kg/m^2^)**	26.4±3.1	24.9±3.3	25.7±3.3	0.17^b^
**Comorbidities**				
Hypertension	13	14	27 (79.4)	1.00^a^
Smoke	13	9	22 (64.7)	0.28^a^
Myocardial infarction	10	8	18 (52.9)	0.52^a^
Diabetes mellitus	6	8	14 (41.2)	0.73^a^
Dyslipidemia	8	6	14 (41.2)	0.73^a^
**EUROSCORE II (%)**	0.73±0.18	0.79±0.17	0.76±0.14	0.34^b^
**Number of bypass graftings (n)**	2.7±0.9	2.7±0.4	2.7±0.6	0.68^c^
**CPB time (min)**	84.2±35.9	77.6±20.8	80.2±28.2	0.88^c^
**Aortic clamp time (min)**	59.2±25	55±13.7	57.5±20.3	0.54^b^
**Surgery time (min)**	225.2±44.6	243.1±60.3	233.2±53.8	0.30^b^
**Intubation time (hours)**	14±7.8	14.3±6.1	14.2±7	0.60^c^
**ICU stay (days)**	3.2±1.2	3.3±0.9	3.3±1	0.57^c^
**Hospital stay (days)**	6.3±1.2	7.5±2.7	6.9±2.1	0.10^c^

BMI=body mass index; ICU=intensive care unit;

a Fisher's exact test.

b Student's t test.

c Mann-Whitney test. Quantitatives variables showed as mean ± standard deviation.

Intubation time, length of stay in ICU and hospital were not significantly different between the groups, with an average duration of 14.27 hours, 3.31 days and 6.92.1 days, respectively (Table 1).

Spirometry results assessed preoperatively and at hospital discharge are shown in Table 2. In both groups, there was a significant decrease in FVC, FEV1 and PEF at hospital discharge compared with the values obtained in preoperative period.

**Table 2:** Pulmonary function at baseline and after early resistance exercise and control groups.

	**Intervention**	**Control**	***P***
**FVC (%)**			
Preoperative	77.7±13.3	81.7±16.9	0.53
Hospital discharge	49.7±13.3	58.3±13.9	0.09
*P*	< 0.0001	0.0004	
**FEV1 (%)**			
Preoperative	83.9±14.8	89±15.3	0.65
Hospital discharge	54.2±14.8	61.8±15.7	0.14
*P*	< 0.0001	0.0002	
**FEV1/FVC (%)**			
Preoperative	108.8±8.3	107.2±12.9	0.94
Hospital discharge	110.6±4.9	106.7±9.7	0.13
*P*	0.57	0.53	
**PEF (%)**			
Preoperative	64.9±18.5	60±22.2	0.45
Hospital discharge	44.5±14.8	47.4±16.2	0.29
*P*	0.002	0.04	

FVC=forced vital capacity; FEV_1_=forced expiratory volume in the first second; FEV_1_/FVC=forced expiratory coefficient in the first second; PEF=peak expiratory flow. Data showed as mean ± standard deviation. Mann-Whitney test.

**Fig. 2:**
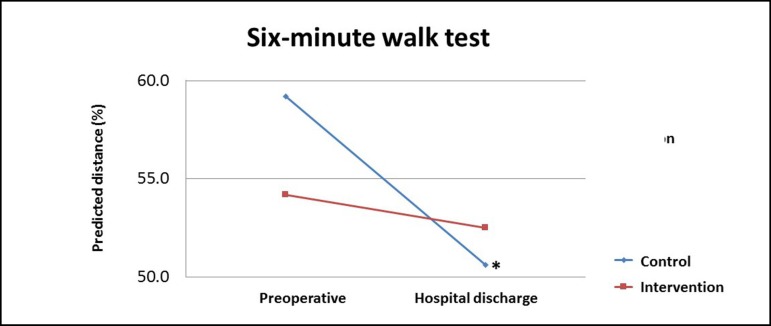
Functional capacity pre-exercise, and post-exercise in the intervention and control groups. Mann-Whitney test.

Intra-group analysis indicated that the control group subjected to conventional physical therapy showed a significant decrease (*P*=0.016) in the predicted distance on the 6MWT when comparing preoperative and hospital discharge assessments. However, the intervention group submitted to resistance exercise maintained the predicted distance on the 6MWT (Table 3 and Figure 2).

**Table 3:** Intra and intergroups analysis of mean predicted distance of six minute walk test in patients undergoing CABG.

	**Preoperative**	**Hospital discharge**	***P***
**Control (%)**	59.2±11.1	50.6±9.9	0.016
**Intervention (%)**	54.1±22.7	52.5±15.5	0.42
***P***	0.18	0.96	

Data showed as mean ± standard deviation. Mann-Whitney test.

*P=0.016 between preoperative and hospital discharge in control group.

## DISCUSSION

Patients undergoing cardiac surgery may present clinical and functional disorders, highlighting pulmonary dysfunction, which leads to decreased lung volume and lung compliance, impaired respiratory function and increased respiratory effort. Decrease in lung volumes and capacities contributes to alterations in gas exchange, resulting in hypoxemia and decreased diffusion^[15]^.

A previous study that compared pulmonary outcomes in patients undergoing CABG indicated that on-pump surgery worsened lung compliance, gas exchange and prolonged intubation time^[16]^. In this regard, all patients in our study underwent on-pump surgery and, therefore, pulmonary dysfunction is expected.

Concerning to respiratory mechanics, our results showed a significant decrease in lung volume and capacity after CABG, in both groups, as other studies in this population^[15,17]^. Oliveira et al.^[18]^ suggested that the decrease in FVC and FEV_1_ occurs by increased respiratory effort, shallower breathing due pain and decreased chest expansion secondary to sternotomy and surgical manipulation, resulting in restrictive ventilatory dysfunctions after surgery.

Decreased FVC and FEV_1_ after cardiac surgery affects coughing and sputum clearance, which may lead to obstruction of small airways, predispose the occurrence of micro-atelectasis and reduction of oxygenation, increasing hospital stay^[19]^.

Exercise programs are essentials for patients undergoing cardiac surgery^[20]^. El-Ansary et al.^[21]^ relate that, although pulmonary dysfunction is common in postoperative period, musculoskeletal disorders are underdiagnosed after cardiac surgery, with an incidence between 40% and 80%^[4]^.

Recently, Gonçalves et al.^[9]^ reported that patients showed good tolerance during the practice of resistance exercise and it was considered safe due to lack of complications or cardiovascular events. These authors related as benefits of resistance exercise in cardiac patients: health improvement, control of risk factors and functional capacity increase. However, it is necessary to emphasize the importance of specific and individual evaluations prior to exercise prescription.

We found decrease in predicted distance walked in 6MWT between preoperative period and hospital discharge, as expected. However, patients who performed resistance exercise maintained their functional capacity when compared to control group.

Kawauchi et al.^[20]^ compared two physical therapy programs, involving a control group (routine protocol) and a training group, submitted to resistance exercise after cardiac transplantation, starting right after extubation. These authors observed similar results in both groups, with improvement in pulmonary function and functional capacity, concluding that the novel treatment program was as beneficial as routine program.

We evaluated the effects of resistance exercise applied early after CABG patients, starting the protocol in the first postoperative day. Researches about this topic, especially, concerning early resistance exercise in this population are scarce, which difficult comparisons of our results, emphasizing the importance of this study.

### Limitations

Some patients presented pulmonary dysfunction and/or complications, requiring noninvasive or invasive ventilation for a long period or even surgical reintervention. Furthermore, peripheral muscle strength was not assessed.

## CONCLUSION

Our results indicate that resistance exercise, applied early, may promote maintenance of functional capacity on CABG patients, having no impact on pulmonary function when compared to conventional physical therapy.

**Table:** 

**Authors' roles & responsibilities**	
NNPSX	Design and drawing of the study; implementation of projects and/or experiments; writing of the manuscript or critical review of its contents; final approval of the manuscript
DLB	Design and drawing of the study; implementation of projects/experiments; analysis/interpretation of data; statistical analysis; manuscript writing or critical review of its contents; final approval of the manuscript
ROL	Execution of operations/experiments
MGBS	Execution of operations/experiments; manuscript writing or critical review of its contents; final approval of the manuscript
LNS	Execution of operations/experiments; final approval of the manuscript
MAGC	Execution of operations/experiments; final approval of the manuscript
TEPB	Execution of operations/experiments; final approval of the manuscript
VJSN	Design and drawing of the study; analysis/interpretation of data; writing of the manuscript writing or critical review of its contents; final approval of the manuscript
